# Guanylyl cyclase/natriuretic peptide receptor-A: Identification, molecular characterization, and physiological genomics

**DOI:** 10.3389/fnmol.2022.1076799

**Published:** 2023-01-04

**Authors:** Kailash N. Pandey

**Affiliations:** Department of Physiology, School of Medicine, Tulane University Health Sciences Center, New Orleans, LA, United States

**Keywords:** natriuretic peptides, particulate guanylyl cyclase receptor-A, structure–function relationship, genomics, gene-targeting, cyclic-GMP signaling

## Abstract

The natriuretic peptides (NPs) hormone family, which consists mainly of atrial, brain, and C-type NPs (ANP, BNP, and CNP), play diverse roles in mammalian species, ranging from renal, cardiac, endocrine, neural, and vascular hemodynamics to metabolic regulations, immune responsiveness, and energy distributions. Over the last four decades, new data has transpired regarding the biochemical and molecular compositions, signaling mechanisms, and physiological and pathophysiological functions of NPs and their receptors. NPs are incremented mainly in eliciting natriuretic, diuretic, endocrine, vasodilatory, and neurological activities, along with antiproliferative, antimitogenic, antiinflammatory, and antifibrotic responses. The main locus responsible in the biological and physiological regulatory actions of NPs (ANP and BNP) is the plasma membrane guanylyl cyclase/natriuretic peptide receptor-A (GC-A/NPRA), a member of the growing multi-limbed GC family of receptors. Advances in this field have provided tremendous insights into the critical role of *Npr1* (encoding GC-A/NPRA) in the reduction of fluid volume and blood pressure homeostasis, protection against renal and cardiac remodeling, and moderation and mediation of neurological disorders. The generation and use of genetically engineered animals, including gene-targeted (gene-knockout and gene-duplication) and transgenic mutant mouse models has revealed and clarified the varied roles and pleiotropic functions of GC-A/NPRA *in vivo* in intact animals. This review provides a chronological development of the biochemical, molecular, physiological, and pathophysiological functions of GC-A/NPRA, including signaling pathways, genomics, and gene regulation in both normal and disease states.

## Introduction

A pioneering and innovative discovery by de Bold and colleagues four decades ago, found natriuretic and diuretic activity in the heart atrium extract, leading to the purification and characterization of atrial natriuretic factor/peptide (ANF/ANP) (de Bold et al., 1981). This discovery revealed a new natriuretic peptide (NP) hormone family and established that the heart is an endocrine organ (de Bold et al., 1981; de Bold, 1985; Pandey, 2005; Goetze et al., 2020). ANF/ANP exhibits diuretic, natriuretic, vasorelaxant, neurotransmission, antimitogenic, and anti-inflammatory responses directed largely toward the reduction of blood pressure (BP) and protection against renal and cardiovascular disorders (Brenner et al., 1990; Levin et al., 1998; Pandey, 2005; Ellmers et al., 2007; Pandey, 2008). After the discovery of ANP, other members of this family were isolated and characterized, including brain natriuretic peptide (BNP), C-type natriuretic peptide (CNP), *Dendroaspis* natriuretic peptide or D-type NP (DNP), and urodilatin (URO) (Goetz, 1990; Stingo et al., 1992; Suga et al., 1992; Lisy et al., 1999). All NPs show similar biochemical, structural, and pharmacological characteristics, with a common 17-amino acid disulfide-bonded ring. Interestingly, each member of the NP hormone family seems to be derived from a separate gene (Rosenzweig and Seidman, 1991). These peptides bind to different cognate receptors, exhibit distinct biological functions, and have varying sites of synthesis (Koller et al., 1992; Levin et al., 1998; Venugopal, 2001; Sharma, 2002; Pandey, 2005; Lee and Burnett Jr., 2007). ANP and BNP are predominantly produced in the cardiac atrium and ventricle, released in the plasma, and exhibit a high variation in sequence structure, whereas CNP is mainly synthesized in the brain and endothelial cells and is highly preserved across the species. DNP is predominantly synthesized in the venom of the green mamba (*Dendroaspis angusticeps*), and URO is produced in the kidney and secreted in the urine (Goetz, 1990; Lisy et al., 1999).

ANP plays a much wider and more significant role, particularly in hypertension and cardiovascular diseases. Both pro-ANP and pro-BNP genes (*Nppa* and *Nppb*) are also expressed in extra-cardiac tissues and cells, which seem to act in endocrine, autocrine, paracrine, and/or neurocrine manners (Vollmer and Schulz, 1990; Pandey and Orgebin-Crist, 1991; Huang et al., 1992; Gutkowska et al., 1993; Pandey, 2005). ANP targets the inhibition of aldosterone secretion from the adrenal glands (Atarashi et al., 1984; DeLean et al., 1984; Goodfriend et al., 1984), release of renin from the kidney (Burnett et al., 1984; Shi et al., 2001), and vasopressin release from the posterior pituitary (Obana et al., 1985). ANP also stimulates the release of testosterone from normal Leydig cells (Mukhopadhyay et al., 1986; Pandey et al., 1986), luteinizing hormone from the anterior pituitary gland (Obana et al., 1985; Horvath et al., 1986; Samson, 2004), and progesterone from granulosa-luteal cells (Pandey et al., 1987). BNP displays functions similar to ANP, but BNP also acts as a neurohormone and is preserved in the transient receptor potential vanilloid-1 (TRPV-1) in response to itch-inducing factors (Mishra and Hoon, 2013). DNP consists of 38 amino acid residues, however, its function has not yet been clearly established (Schweitz et al., 1992; Lisy et al., 1999). URO is a 32-residues peptide hormone similar to the carboxyl-terminal sequence of pro-ANP, which was isolated and characterized from urine (Schulz-Knappe et al., 1988; Feller et al., 1990). It is believed that URO is largely synthesized in the kidneys but mostly absent in the circulation (Saxenhofer et al., 1990; Goetz, 1991). Interestingly, URO is very resistant to proteolysis by endopeptidases and has an important role in the regulation of kidney function; more specifically, it controls the excretion of sodium and water, much like ANP and BNP (Goetz, 1990; Emmeluth et al., 1992).

The pharmacological and physiological functions of NPs is elicited through the binding of cognate plasma membrane receptor proteins. Three distinct subtypes of NPs receptor proteins have been identified and characterized: guanylyl cyclase (GC)/NP receptor-A (GC-A/NPRA), GC/NP receptor-B (GC-B/NPRB), and NP receptor-C (NPRC), encoded by specific genes, including *Npr1*, *Npr2*, and *Npr3*, respectively (Pandey, 2008). Both ANP and BNP activate GC-A/NPRA, also known as GC-A receptor, which responds to hormone binding by producing intracellular second messenger cGMP to this receptor molecule. CNP specifically activates GC-B/NPRB, known as GC-B receptor, and also produces a second messenger, cGMP. All three NPs (ANP, BNP, and CNP) invariably bind to NPRC, which lacks an intracellular GC region (Koller et al., 1992; Pandey, 1992; Pandey, 1996; Levin et al., 1998; Pandey, 2005). The prevalence of structurally related NPs and their three distinct receptors suggests that their role in physiological and pathophysiological control of BP, body fluid homeostasis, and metabolic regulation is complex. At the minimum, three distinct subtypes of effector molecules are prevalent: cGMP-dependent protein kinases (PKGs), cGMP-dependent phosphodiesterases (PDs), and cyclic-nucleotide gated ion channels (CNGs), which catalyze and amplify the signaling cascade of NP-specific cognate receptors (Pandey, 2005; Sharma, 2010; Kishimoto et al., 2011; Pandey, 2011).

In essence, GC-A/NPRA acts as the main functional receptor protein for both ANP and BNP; and in a large part, the biological and physiological functions of hormones are discharged by the production of intracellular generation cGMP (Lucas et al., 2000; Sharma, 2002; Pandey, 2005; Pandey, 2008; Sharma, 2010; Pandey, 2011). In mice, the gene-targeting strategy of *Npr1* revealed the hallmark significance of GC-A/NPRA in the regulation of BP and protection against renal and cardiovascular dysfunction (Oliver et al., 1997, 1998; Shi et al., 2003; Vellaichamy et al., 2005; Ellmers et al., 2007; Kishimoto et al., 2011; Pandey, 2011; Das et al., 2012; Vellaichamy et al., 2014; Subramanian et al., 2022). CNP is produced in the endothelial cells, activates NPRB in the neighboring vascular smooth muscle cells (VSMCs), and has a pivotal function in the clinical hypotension of septic shock by decreasing venous return (Suga et al., 1992, 1993; Hama et al., 1994). The current review briefly summarizes previous studies and salient discoveries in the NPs hormone family and their receptors with a major emphasis on GC-A/NPRA, including signaling mechanisms, structure–function composition, and roles in the physiology and pathology of health and disease.

## Identification, characterization, and biochemical properties of NP receptors

Initially, to identify NP receptors, specific ^125^I-ANP binding was performed using plasma membrane preparations of different tissues and intact cells (Misono et al., 1985; Pandey et al., 1986, 1988; Leitman et al., 1988). Initial cross-linking and photoaffinity studies showed that the molecular weight (M_r_) of ANP receptors varies widely, ranging from 60-to 180-kDa. They were classified and characterized by sodium dodecyl sulfate polyacrylamide gel electrophoresis (SDS-PAGE) and autoradiography using various tissues and cells (Misono et al., 1985; Schenk et al., 1985; Vandlen et al., 1985; Yip et al., 1985; Meloche et al., 1986; Pandey et al., 1986, 1987, 1988). Later, photoaffinity and cross-linking methods showed a single protein band of ANP receptor with an apparent M_r_ of 135-to 140-kDa in plasma membrane preparations of various tissue and cell types (Pandey et al., 1987; Takayanagi et al., 1987; Leitman et al., 1988; Pandey et al., 1988; Pandey, 1993). An additional protein band of 70-kDa was also identified using affinity cross-linking and photoaffinity labeling techniques in the isolated plasma membranes and intact cells (Hirose et al., 1985; Schenk et al., 1985; Vandlen et al., 1985; Meloche et al., 1986; Leitman et al., 1988; Pandey et al., 1988). Concurrently, ^125^I-ANP high-affinity binding sites of 130-to 180-kDa of ANP receptors were co-purified with GC activity (Paul et al., 1987; Takayanagi et al., 1987; Marala and Sharma, 1988; Meloche et al., 1988). A 70-kDa ANP receptor which did not display GC activity was also isolated and characterized (Schenk et al., 1987; Takayanagi et al., 1987; Pandey et al., 1988). Based on the biological activity of the different lengths of ANP molecules, NPs receptor are classified into 130-to 180-kDa proteins (biologically active) and 60 to 68-kDa protein molecule (clearance) receptor, which was unable to produce cGMP (Pandey, 2005).

Later, three distinct subtypes of ANP receptors were identified and characterized by using the criteria, including binding characteristics, cross-linking and photoaffinity labeling techniques, and capacity to generate intracellular cGMP, which were found to be specific to different cell and tissue types (Leitman et al., 1988; Pandey et al., 1988). Three distinct NP receptor subtypes were identified, characterized, and classified in multiple cells and tissues, including non-reducible 135-kDa, reducible 140-kDa, and non-reducible 70-kDa protein bands (Pandey et al., 1988). In VSMCs, two specific binding sites of the 70-kDa and 140-kDa bands were identified, while under reducing conditions, the 70-kDa receptor band persisted; however, the 140-kDa protein band was disappeared and reduced to 70-kDa. Non-iodinated or unlabeled 28-amino-acid full-length ANP abolished both 70-kDa and 140-kDa bands; however, truncated ANF (c-ANF or AP I) inhibited only the 70-kDa receptor band. The renal extracts and established Maiden-Darby canine kidney (MDCK) cells showed 70-kDa and 120-kDa receptor bands, but Leydig tumor (MA-10) cells and neuroblastoma glial cells yielded just a 135-kDa receptor band, which was eliminated with full-length ANP but not with truncated AP-1 (Pandey et al., 1986; Pandey and Inagami, 1988). VSMCs contained approximately 90% low M_r_ 66–70-kDa protein bands (Pandey et al., 1987; Leitman et al., 1988; Pandey et al., 1988). Interestingly, adrenal glomerulosa cells, MDCK, and MA-10 cells contained the non-reducible 125-to 180-kDa ANP receptor bands and showed similar characteristics both biochemically and pharmacologically. Biochemical, cellular, molecular, and immunohistochemical studies suggested that ANP receptors seem to be widely distributed in different cells and tissue types, with pleiotropic functional characteristics in the renal, cardiac, vascular, and neural systems (Brenner et al., 1990; Levin et al., 1998; Pandey, 2005; Pandey, 2018).

## Molecular cloning and determination of primary structure of NP receptors

Molecular cloning of cDNAs from mouse, rat, and humans deduced the primary structure of three subtypes of NP receptors (NPRs), including: GC-A/NPRA, GC-B/NPRB, and NPRC, which constitute the NP receptor family (Fuller et al., 1988; Chang et al., 1989; Chinkers et al., 1989; Lowe et al., 1989; Pandey and Singh, 1990; Marala et al., 1992). The primary structural topology of the extracellular ligand-binding domain (LBD) of NPRA and NPRB exhibit almost 45% sequence homology. The protein kinase-like homology domain (protein-KHD) contains 280 amino acid residues that follow the transmembrane domain (TD) region of the receptors; however, the intracellular carboxyl-terminus portion of GC-A/NPRA and GC-B/NPRB contains 250 amino acids, constituting GC catalytic domain (GCCD) (Pandey and Singh, 1990; Drewett and Garbers, 1994; Zhang et al., 1997). The GCCD enzymatic region showed the greatest homology, with GC-A/NPRA and GC-B/NPRB sharing almost 80% sequence identify while protein-KHD had only 60% sequence identify among both GC-A/NPRA and GC-B/NPRB. Over 80% of the conserved amino acid residues present in all protein kinases seem to be found in the protein-KHD of GC-A/NPRA and GC-B/NPRB receptors (Hanks et al., 1988; Koller et al., 1992). But the functional significance of protein-KHD remains unclear. Based on amino acid sequence comparisons, there seems to be over 60% sequence identity among GC-A/NPRA and GC-B/NPRB receptor proteins. Sequence homology data have revealed that the intracellular regions of these two GC receptors appear to be highly conserved, reaching almost 80% sequence identity. However, the extracellular LBD of these two GC receptors seem to have only 43% sequence similarity.

Five of the six cysteine residues of both GC receptors (NPRA and NPRB), are found in extracellular domains in the same relative positions (Pandey and Singh, 1990). The intracellular region of GC-B/NPRB does not contain any putative glycosylation sites, and out of the all seven N-like glycosylation sites present in the extracellular domain of both receptors, only two are conserved across species (Schulz et al., 1989; Pandey and Singh, 1990; Drewett and Garbers, 1994). The nucleotide sequence in the GCCD of GC-A/NPRA and GC-B/NPRB is more highly conserved than in the protein-KHD region, whereas NPRC comprises a large extracellular LBD region of 496-amino acid residues, a single TD region, and a short 37-amino acid intracellular cytoplasmic tail, which does not exhibit any sequence homology with other known membrane receptor proteins. Extracellular LBD of NPRC contains only 30% sequence homology to GC-A/NPRA and GC-B/NPRB. Characteristically, NPRC possesses a very low specificity for different lengths of ANP peptides compared to GC-A/NPRA and GC-B/NPRB receptors (Fuller et al., 1988; Bovy, 1990; Khurana and Pandey, 1993). The clearance nomenclature of NPRC was given only by the default hypothesis; however, NPRC is also thought to display some biological functions (Pandey, 1992; Matsukawa et al., 1999; Palaparti and Anand-Srivastava, 2000; Zhou and Murthy, 2003).

## Topology and domain structure of GC-A/NPRA and other GC receptors

**Figure 1:**
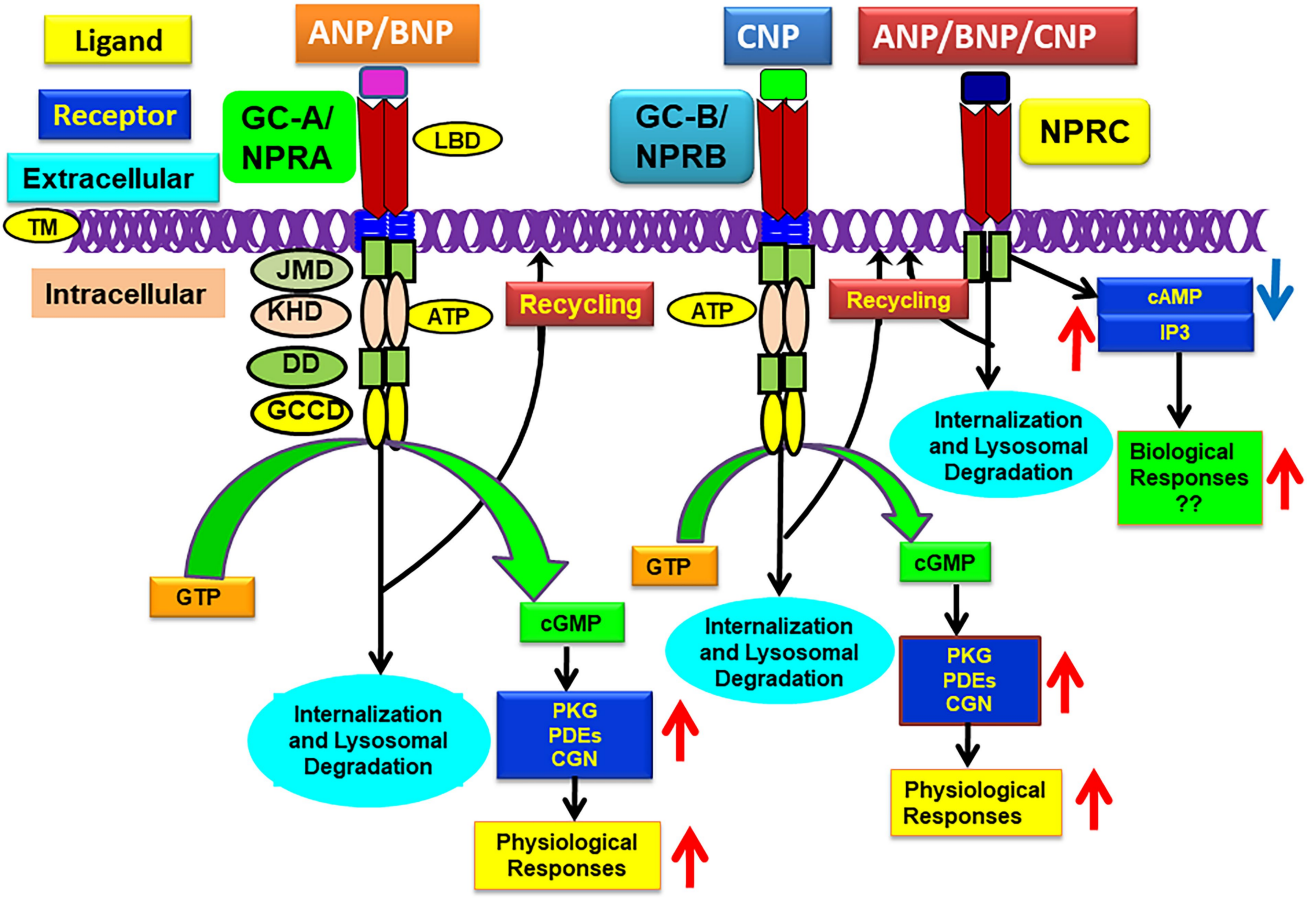
Schematic representation of ligand-dependent activation and physiological functions of GC-A/NPRA, GC-B/NPRB, and NPRC. ANP and BNP binding to amino-terminal domain activates GC-A/NPRA, which leads to enhanced production of intracellular second messenger cGMP with stimulation of PKG, PDFs, and CNG that activate ANP-dependent cellular and physiological responsiveness. CNP binds and activates GC-A/NPRB and also produces cGMP and physiological responsiveness. All three NPs activate NPRC that lead to some biological actions. ANP, atrial natriuretic peptide; BNP, brain natriuretic peptide; CNP, C-type natriuretic peptide; GC-A/NPRA, guanylyl cyclase-A/natriuretic peptide receptor-A; GC-B/NPRB, guanylyl cyclase-B/natriuretic peptide receptor-B; NPRC, natriuretic peptide clearance receptor; LBD, ligand binding domain; TM, transmembrane domain; KHD, protein-kinase like homology domain; DD, dimerization domain; GCCD, guanylyl cyclase catalytic domain; IP_3_, inositol trisphosphate. Adopted with modification from reference Pandey, K. N. 2015, Membranes 5, 253–287.

Among the members of GC family of membrane receptors, GC-A/NPRA represents a biologically active NP receptor molecule, largely prevalent in peripheral tissues and cells which elicits most of ANP and BNP’s known actions (Drewett and Garbers, 1994; Levin et al., 1998; Sharma, 2002; Pandey, 2005). In contrast, GC-B/NPRB is largely localized in vascular tissues and in the central nervous system (CNS) and it mediates the action of CNP, which also generates the production of second messenger, cGMP (Schulz et al., 1989; Duda and Sharma, 1995; Lowe, 1997; Lucas et al., 2000). Both GC-A/NPRA and GC-B/NPRB constitute an overall domain organization and topology structure much like that of other GC receptors. Both GC-A/NPRA and GC-B/NPRB consist of generally five separate domains, including LBD, a single membrane spanning TD, intracellular cytoplasmic protein-KHD, dimerization domain (DD), and enzymatic GCCD (Schulz et al., 1989; Pandey and Singh, 1990; Drewett and Garbers, 1994; Sharma, 2002; Pandey, 2005). One polypeptide molecule of transmembrane GC receptors contains a single GC catalytic active site, while the structural predicted data indicated that two-polypeptide chains are required to functionally activate GC-A/NPRA (Wilson and Chinkers, 1995; Yang and Garbers, 1997; Labrecque et al., 1999; van den Akker et al., 2000). Modeling data also predicted that the dimerization domain of GC-A/NPRA is embedded between the GCCD and protein-KHD catalytic regions and forms an amphipathic alpha helix structure of the receptor molecule (Garbers and Lowe, 1994). The sequences of different domains of GC-A/NPRA are conserved among mammalian species, including mouse, rat, and human (Chinkers et al., 1989; Lowe et al., 1989; Pandey and Singh, 1990; Marala et al., 1992; Garg et al., 2002). Based on sequence comparison analysis data, the protein-KHD of GC-A/NPRA is related more closely to the receptor tyrosine kinases (RTKs) than the serine/threonine kinases. The protein-KHDs of GC-A/NPRA and GC-B/NPRA have been suspected of exhibiting an important role in transmitting the ligand-induced signals of these receptor proteins (Chinkers and Garbers, 1989; Duda et al., 1993a; Sharma, 2002, 2010; Pandey, 2008). An intervening step may be necessary to activate the catalytic process of GCCD involving protein-KHD of GC-A/NPRA (Goraczniak et al., 1992; Koller et al., 1993; Sharma, 2002); however, the activation of GC-A/NPRA and GC-B/NPRB receptors probably requires ATP, which serves as an intracellular allosteric regulator to protein-KHD (Kurose et al., 1987; Chinkers et al., 1991; Larose et al., 1991; Duda et al., 1993a; Wong et al., 1995). The deletion of C-terminal sequences of GC-A/NPRA resulted in a truncated protein product that showed binding specificity to ANP but did not exhibit the GC activity of the receptor molecule (Thorp and Morkin, 1990; Koller et al., 1992; Pandey and Kanungo, 1993; Pandey et al., 2000). Crystal structure modeling analysis of adenylyl cyclase II C2 (ACII C2) domain indicated that the catalytic enzymatic active sites of GC receptors and ACs seem to be poorly related to the structural topology of GC-coupled receptors (Liu et al., 1997; Zhang et al., 1997; Sunahara et al., 1998; Tucker et al., 1998). The GCCD enzymatic active site of GC-A/NPRA seems to include a region of 31-amino acid residue sequence at the carboxyl-terminus end (974–1,004 residues) of the receptor. In fact, the members of the GC family of receptor protein constitute a single GCCD enzymatic business end for each polypeptide chain and exhibit catalytic activity as homodimers, which generates the second messenger cGMP and activates physiological signaling cascades (Figure 1).

Guanylyl cyclase C (GC-C) is also a member of the GC receptor family, which contains topology of domain structure, including extracellular LBD, TD, protein-KHD, and GCCD similar to GC-NPRs. However, GC-C is predominantly expressed and largely found on the apical surface of intestinal cells and activated by gastrointestinal peptides guanylin and uroguanylin and by heat-stable enterotoxin (ST) produced by *Escherichia coli* (Schulz et al., 1990; Currie et al., 1992; Hamra et al., 1993; Arshad and Visweswariah, 2012; Cappelli et al., 2019; Bose et al., 2020). GC-C is also expressed in the kidney and enhances the excretion of Na^+^ and water. Paradoxically GC-C KO mice seem to exhibit normal gastrointestinal function but were found to be defiant to ST-activated diarrhea (Schulz et al., 1997; Sindice et al., 2002). Both guanylin and uroguanylin are thought to function in an endocrine manner (Forte et al., 2000; Carrithers et al., 2002). Among the GC family of receptors, besides NPRs and GC-C, several other members have been identified, including GC-D, GC-E, GC-F, GC-G, and Ret-GC or ROS-GC. However, the details of these receptors will not be discussed in this review; nevertheless, all members of GC-family of receptors transmit the signal by generating the second messenger cGMP.

## Signal transduction mechanisms and intracellular signaling of GC-A/NPRA

ANP greatly increases the accumulation of intracellular cGMP levels and decreases the concentration of cAMP by stimulating the specific PDEs in target tissues and cells in a dose-and time-dependent manner (Waldman et al., 1984; Pandey et al., 1985; Tremblay et al., 1985; Pandey et al., 1986; Levin et al., 1998; Pandey, 2005). However, the inhibitory effects of ANP on cAMP levels seem to act indirectly, probably by involving cGMP-dependent PDEs (Pandey, 2005; Sharma, 2010). Earlier studies suggested that ANP binding to GC-A/NPRA alone might not be sufficient to stimulate the maximum levels of GC activity, but it may also require ATP (Kurose et al., 1987; Chinkers et al., 1991; Goraczniak et al., 1992). The nonhydrolyzable analogs of ATP mimics the effects of ANP, and ATP might act by allosteric regulation of GC activity of GC-A/NPRA. The deletion and/or point mutations in GC receptors lacking a protein-KHD region did not show responsiveness of either ANP or ATP to activate GC activity of these receptors (Chinkers and Garbers, 1989; Koller et al., 1992; Duda and Sharma, 1995; Sharma, 2002). The mutant receptor was thought to be independent of any ANP requirement; however, it showed the ability to bind the ligand and exhibited 100-fold excess GC activity compared with wild-type (WT) receptor protein (Chinkers and Garbers, 1989; Koller et al., 1992). ATP binding, possibly to protein-KHD, causes a structural conformational change to ease the protein-KHD autoinhibition of GC enzymatic catalytic region during the ligand-induced signaling cascade of GC-A/NPRA (Lowe, 1997; Foster and Garbers, 1998; Garbers, 1999). This model has been challenged by other investigators, who proposed that the deletion of the protein-KHD of GC-A/NPRA was unable to enhance the basal GC activity of the receptor (Goraczniak et al., 1992; Duda et al., 1993b; Sharma, 2002, 2010); however, those previous studies agreed that ATP is likely necessary for the hormone-dependent maximum activation of GC-coupled receptors. The authors further suggested that the interactive action of ATP with protein-KHD enhances the effectiveness of the ligand-induced signaling mechanisms of GC-A/NPRA with enhanced accumulation of intracellular second messenger cGMP.

Earlier, it was predicted that after ligand binding, ATP interacts with protein-KHD, which increases the production of intracellular cGMP without affecting the affinity of the substrate to the receptor (Kurose et al., 1987; Chang et al., 1990; Duda et al., 1991; Gazzano et al., 1991; Wong et al., 1995; Sharma, 2002). Later studies suggested that ATP binding to protein-KHD seems to be critical for the effector-coupling of GC-coupled receptor proteins (Goraczniak et al., 1992; Duda et al., 1993a; Sharma, 2002, 2010). Those studies further indicated that the glycine-rich motif G-R-G-S-N-Y-G in the protein-KHD was important for ATP binding and activation of the ANP/NPRA signaling cascade (Duda et al., 1991, 1993a; Goraczniak et al., 1992). The consensus sequence of the glycine-rich motif was named the ATP-regulatory module (ARM) of the GC receptors (Goraczniak et al., 1992; Duda et al., 1993a; Sharma, 2002). Site-directed mutagenesis experiments suggested that the middle Gly residue in the glycine-rich consensus motif was crucial for the ATP-binding site and for the signal transduction cascade of GC-coupled receptors (Duda et al., 1993a; Sharma, 2002). Tight regulatory control of the receptor signal seems to be essential for the intracellular formation of the second messenger cGMP (Sharma et al., 1994; Sharma, 2002). ATP-binding to the protein-KHD has also been proposed to cause GC activation and induces a low-affinity shift that might release the bound ligand from the receptor molecule; however, this remains to be experimentally tested to whether modulates the signaling cascade of GC-coupled receptors (Jewett et al., 1993).

Crystallographic modeling data have shown that in the absence of ligand, LBD of GC-A/NPRA is self-associated to form a homodimer molecule (van den Akker et al., 2000). The binding of ligand to the receptor induces molecular conformational alterations and/or coordinated structural changes in the protein-KHD, which likely leads to ATP binding to enhance the signaling cascade (Huo et al., 1999; Misono et al., 2005). However, it is unclear whether native target cells also exhibit the dimeric nature of the receptor molecules. Evidence suggests two possible dimer pairs exist in the crystal packaging of the extracellular LBD of GC-A/NPRA; the tail-to-tail and head-to-head dimers seem to be associated with the membrane-distal and proximal subdomains of the receptor (Qiu et al., 2004), respectively. The tail-to-tail dimer of GC-A/NPRA may be required for ligand-induced signaling of this receptor protein (van den Akker et al., 2000; Misono et al., 2005). Moreover, the crystallographic structure of NPRC has also indicated that a dimerized form of this receptor seems to be essential in head-to-head configuration with bound ligand-receptor complex (He et al., 2001, 2005). The head-to-head dimer of NPRC seems to represent the inactive state of the receptor, whereas the tail-to-tail dimer may represent its hormone-activated state (He et al., 2001, 2005; van den Akker, 2001; Misono et al., 2005). The ligand-mediated activation of GC-A/NPRA may stabilize the membrane-distal dimer interface, indicating that ligand binding confers the GC-A/NPRA dimer interface interactive site of the receptor (DeLean et al., 2003). Chemical modifications and site-directed mutagenesis experiments have indicated that the head-to-head dimer structure confers with the physiological dimer of GC-A/NPRA (Qiu et al., 2004). The disruption of disulfide cysteine bonds in the juxtamembrane hinge region (JMHR) results in constitutive activation of the receptor, indicating that the JMHR might play a crucial role in the signal transduction and receptor activation mechanisms of GC receptors (Huo et al., 1999; Misono et al., 2005, 2011).

The heterogeneity and diverse cellular distribution of NP receptors indicates that different mechanisms might govern the activation of signaling and function of GC receptors in target cells (Pandey et al., 2000; Sharma, 2002; Pandey, 2005; Sharma, 2010; Kishimoto et al., 2011; Pandey, 2011). ANP stimulates the generation of inositol triphosphates (IP_3_) at very low concentrations in cultured MA-10 cells and VSMCs (Resink et al., 1988; Hirata et al., 1989; Khurana and Pandey, 1994; Khurana and Pandey, 1995; Pandey, 2014); however, ANP stimulated the generation of IP_3_ in the renal inner medullary collecting duct cells (RIMCDs) and MA-10 Leydig tumor cells at lower concentrations, while at higher hormone concentrations, it inhibited the formation of these metabolites, simultaneously increasing the accumulation of intracellular cGMP levels (Teitelbaum et al., 1990; Berl et al., 1991; Khurana and Pandey, 1995; Khurana and Pandey, 1996; Pandey, 2005; Pandey, 2014). Depending on cell type, ANP inhibits both the enzymatic activity and autophosphorylation of protein kinase C (PKC) (Pandey, 1989; Pandey, 1994; Kumar et al., 1997; Pandey, 2008). Furthermore, ANP stimulated potassium channels through the activation of PKGs, which requires ATP (White et al., 1993). Our studies have shown that GTPγS synergistically enhanced ANP’s effect on the GC catalytic activity of GC-A/NPRA (Khurana and Pandey, 1995), but the antibodies against the subunits of G-proteins (Gsα and Giα) did not affect GC activity, although Goα antibodies blocked the agonist-stimulated GC catalytic activation of GC-A/NPRA (Khurana and Pandey, 1994; Pandey, 2005).

GC-A/NPRA seems to be present in the phosphorylated state. ANP causes a decrease in the phosphate content and thus dephosphorylation, in turn reducing ANP-dependent GC activity and desensitizing the receptor protein (Potter and Garbers, 1992; Potter and Garbers, 1994). Earlier findings have indicated that ANP seems to stimulate the phosphorylation of GC-A/NPRA (Ballerman et al., 1988; Pandey, 1989; Duda and Sharma, 1990; Larose et al., 1992). It was later suggested that ANP stimulates the phosphorylation of NPRA at serine and threonine residues in the protein-KHD region of the receptor that was considered essential for receptor activation (Foster and Garbers, 1998; Potter and Hunter, 1998). Those studies indicated that phosphorylation at serine and threonine sites induces the desensitization of GC-coupled receptors. In fact, the activation of GC-A/NPRA could also be attenuated by agents such as certain growth factor, including epidermal growth factor (EGF) and platelet-derived growth factor (PDGF) and pressure hormones, such as endothelin, vasopressin, and ANG II, which decrease the responsiveness of GC-A/NPRA (Haneda et al., 1991; Yasunari et al., 1992; Potter and Garbers, 1994; Kumar et al., 1997; Pandey et al., 2000; Sharma et al., 2002; Garg and Pandey, 2003; Tripathi and Pandey, 2012; Alicic et al., 2018; Arise et al., 2020). The agonist-dependent activation of PKC by phorbol ester decreased GC catalytic activity of NPRA (Haneda et al., 1991; Yasunari et al., 1992; Potter and Garbers, 1994; Kumar et al., 1997; Pandey, 2005; Kumar et al., 2017). On the other hand, the desensitization of GC-A/NPRA may be correlated with mechanisms involving receptor phosphorylation (Duda and Sharma, 1990). The mechanism of desensitization of NPRA involving dephosphorylation does not seem to be consistent with G-protein-coupled receptor molecules, which also appear to be desensitized by protein phosphorylation (Zhang et al., 1997; Lefkowitz et al., 1998). Both protein kinases and protein phosphatases seem to be involved in the desensitization mechanisms of GC-A/NPRA, which remain poorly understood. But one study has suggested that PKG seems to phosphorylate the GC-A/NPRA *in vitro* system (Airhart et al., 2003). Previous studies further indicated that after ANP treatment, PKG is recruited to the plasma membrane and enhances the GC catalytic activity of the receptor. Interestingly, PKG seems to translocate in an ANP-mediated manner; however, not in the nitric oxide-mediated system. An ANP-mediated NPRA/PKG mechanism might initiate the cGMP-dependent signaling in the functional regulation of GC-A/NPRA in target cells.

## Endocytosis, intracellular trafficking, and downregulation of GC-A/NPRA

Receptor internalization is a prominent mechanism underlying the concentrated uptake of ligand-receptor complexes for receptor-mediated intracellular signal transduction, neurotransmission, cellular activities, and physiological and pathophysiological functions. We have previously suggested that receptor endocytosis and intracellular signaling of GC-A/NPRA occur concurrently during internalization and subcellular trafficking. During endocytotic process the second-messenger cGMP signals are generated in intact cells (Mani et al., 2015, 2016; Mani and Pandey, 2019). Stoichiometric analyses of the endocytosed ANP/NPRA complexes and metabolic processing showed that the bound hormone-receptor complexes were endocytosed and intracellularly processed, and the metabolized degraded products were ultimately exocytosed and released into culture medium (Pandey et al., 1986; Rathinavelu and Isom, 1991; Pandey, 1993; Pandey et al., 2002; Mani et al., 2015). However, a small population of bound ligand-receptor complexes may escape the degradative lysosomal pathway and recycle back to the plasma membrane, leaving some intact ligands to be released in the cell exterior (Pandey et al., 2002, 2005; Saftig and Klumperman, 2009; Mani and Pandey, 2019). Using MA-10 cells (harboring the native receptor population) and HEK-293 cells (expressing the recombinant receptor molecules), we have shown that after ligand-binding, the bound hormone-receptor complexes of ANP-BNP/GC-A/NPRA are endocytosed, intracellularly processed, and metabolized inside the cell (Pandey et al., 1986, 2000, 2002; Pandey, 1993, 2001; Somanna et al., 2013; Mani et al., 2016). On the other hand, one study indicated that in renomedullary epithelial cells (RMECs), GC-A/NPRA with bound-ligand receptor complex was not processed intracellularly and rapid dissociation of ligand-receptor complexes occurred after ANP binding to GC-A/NPRA, and intact ligand was released into culture medium (Koh et al., 1992). However, since the ligand dissociation was performed using very high amount of cold (unlabeled) ANP, rebinding of the dissociated ligand to the receptor protein can be excluded, making these findings difficult to interpret (Koh et al., 1992). These findings also indicated that RMECs contained multiple receptor populations including GC-A/NPRA and NPRC (Koh et al., 1992). Studies of ligand binding and metabolic processing of ANP involving NPRCs have been reported using VSMCs, which predominantly contain 70-kDa NPRCs and only a small population of GC-A/NPRAs (Hirata et al., 1985; Napier et al., 1986; Pandey et al., 1988; Murthy et al., 1989; Cahill et al., 1990; Nussenzveig et al., 1990; Pandey, 1992, 2005; Cohen et al., 1996).

The antibody-tracking method has indicated that both GC-A/NPRA and GC-B/NPRB were found to be internalized in a ligand-independent manner (Dickey et al., 2011); however, this method only qualitatively determines the internalization kinetics of ligand-receptor complexes. Immunofluorescence confocal microscopy has revealed the visualization of the subcellular trafficking and ligand-dependent endocytosis of GC-A/NPRA tagged with eGFP- (eGFP-GC-A/NPRA) in HEK-293 and murine mesangial cells (MMCs) (Mani et al., 2015, 2016). The internalization of GC-A/NPRB was also demonstrated in hippocampal neurons and glioma cells (Brackmann et al., 2005). Endocytosis, sequestration, and metabolic processing of internalized hormone receptor complexes may all play roles in the downregulation of receptors (Pandey et al., 2002; Pandey, 2010; Pandey, 2015). GC-A/NPRA downregulation has been reported in PC-12 cells that contain endogenous receptors (Rathinavelu and Isom, 1991) and COS-7 and HEK-293 cells transfected with recombinant receptors (Pandey et al., 2000, 2002, 2005). The deletion of carboxyl-terminus regions of GC-A/NPRA suggested that the specific sequence in catalytic GCCD and protein-KHD played a pivotal role in the internalization, sequestration, and metabolic degradation of the receptor protein (Pandey et al., 2000; Pandey, 2015). Interestingly, several studies have indicated that micro-RNA (miR) plays critical roles in the regulatory mechanisms and function of ANP/NPRA system (Arora et al., 2013; Somanna et al., 2013; Wu et al., 2016; Vandenwijngaert et al., 2018; Khurana et al., 2022). Our recent findings have suggested that prolonged ANP treatments along with miR-128 and miR195 of cultured cells expressing high density of GC-A/NPRA caused downregulation of the receptor in a time-and dose-dependent manner (Khurana et al., 2022). Those findings showed that miR-128 and miR-195 caused significant reduction in the protein levels of GC-A/NPRA. We implicated that ligand-mediated mechanisms involving miR-128 and miR-195 might instigate a prominent regulatory role in the regulation and activity of GC-coupled receptor proteins.

Using the confocal microscopy and ^125^I-ANP binding assay, we delineated the significance of dynamin molecule in the trafficking and internalization of GC-A/NPRA utilizing the recombinant HEK-293 cells (Somanna et al., 2018). ANP treatment enhanced the internalization of NPRA in the cell interior, but the process was significantly impaired by the specific inhibitors of clathrin/dynamin, namely monodansylcadaverine (MDC) and chlorpromazine (CPZ) (Somanna et al., 2018). Mutant dynamin also specifically blocked the endocytic vesicle formation and internalization of NPRA (Somanna et al., 2018). Immunofluorescence visualization of GFP-tagged GC-A/NPRA in HEK-293 cells has revealed that endocytic vesicles are formed within 5 min after ANP treatment; however, the process was blocked by the inhibitors of clathrin (MDC and CPZ) and mutant dynamin (Somanna et al., 2018). We further showed that GC-A/NPRA undergoes internalization *via* clathrin-mediated endocytosis, including receptor internalization, signaling, and metabolic degradation. Our previous studies indicated that the internalization of GC-A/NPRA occurred *via* clathrin-dependent pathways following initial cluster formation of receptor-ligand cargo in clathrin-coated pits on the plasma membrane. The blockade of the endocytosis of ligand-receptor complexes by the clathrin inhibitors (MDC and CPZ), provided an efficient and valuable method to facilitate the mechanistic actions of the endocytosis of GCA/NPRA. *Via* confocal microscopy, the formation of endocytic vesicles during the internalization of receptor molecules was revealed. CPZ, MDC, and mutant dynamin all affected both the formation of clathrin-coated pits and the assembly of clathrin (Law et al., 2011; Schwartz et al., 2012; Smani et al., 2012). The role of this receptor in the physiology and pathophysiology of hypertension and cardiovascular homeostasis may be clarified by the fact that the clathrin-mediated endocytic pathway is considered a major route for the internalization of GC-A/NPRA.

Short signal-sequence motifs appear to be essential to the internalization and intracellular trafficking of plasma membrane receptors, which are thought to send the ligand-receptor cargo into trafficking endocytic vesicles (Pandey, 2009; Kozik et al., 2010; Pandey, 2010; Davey et al., 2012; Pandey, 2015). These small motifs comprise a linear array of a short sequence of amino acids containing 2–6 amino acid residues, however, only 2–3 amino acids of which are essential to receptor internalization and intracellular trafficking processes (Bonifacino and Traub, 2003; Davey et al., 2012; Mardones et al., 2013). The sequence motif Gly^920^-Asp^921^-Ala^922^-Tyr^923^ (GDAY) in the C-terminal-region of GC-A/NPRA acts to promote the endocytosis and trafficking processes of NPRA (Pandey et al., 2005). Gly^920^ and Tyr^923^ residues in GDAY direct internalization of GC-A/NPRA, but Asp^921^ provides an acidic environment for GDAY signaling in the intracellular routing and subcellular trafficking processes. Site-directed mutagenesis of Gly^920^ and Tyr^923^ residues to Ala blocked the endocytosis of GC-A/NPRA by 50%; however, no effect on the recycling process was found. The site-directed mutation of Asp^921^ to Ala did not seem to affect receptor endocytosis, but it did potentially prevent the recycling of internalized receptors to the plasma membrane. We have demonstrated that FQQI short amino acid motif also plays a critical role in the endocytosis and subcellular trafficking of GC-A/NPRA (Mani et al., 2016; Mani and Pandey, 2019). Confocal immunofluorescence analyses showed that WT receptor (eGFP-GC-A/NPRA) was rapidly internalized and redistributed into cellular compartments, but the mutant FQQI/AAAA motif markedly inhibited endocytosis, signaling process, and subcellular trafficking of GC-A/NPRA (Mani et al., 2016; Mani and Pandey, 2019). FQQI short sequence motif plays a significant role in maintaining continuous receptor signaling. These findings expanded our knowledge of the cellular and molecular mechanisms of internalization, subcellular trafficking, and concurrent simultaneous signaling of GC-A/NPRA in intact cell.

## GC-A/NPRA regulates renal hemodynamics, blood pressure, and cardiovascular events

The use of gene-targeting methods in mice has yielded novel approaches to decipher the biological and pathophysiological functions of the ablated gene products in intact animals *in vivo* (Takahashi and Smithies, 1999; Kim et al., 2002). Genetic strategies have generated mice carrying gene knockout (KO) or gene duplication, providing proof-of-concept for the physiological and pathophysiological roles of GC-A/NPRA in the regulation of renal and cardiovascular hemodynamic parameters (John et al., 1995; Lopez et al., 1995; Kishimoto et al., 1996; Oliver et al., 1997, 1998; Matsukawa et al., 1999; Pandey et al., 1999; Shi et al., 2003; Vellaichamy et al., 2005; Ellmers et al., 2007). Early studies demonstrated that the ablation of *Npr1* (encoding GC-A/NPRA) led to an increase in BP in *Npr1* KO mice (Oliver et al., 1997; Shi et al., 2001, 2003). Investigations in our laboratory have shown that at birth, loss of NPRA permitted increased synthesis and release of renin in KO mice compared with their WT counterparts; however, in adult mice, ANG II levels, along with circulating and kidney renin contents, were greatly reduced in *Npr1* null mutant KO mice compared to *Npr1* WT mice (Shi et al., 2001). Those studies showed that the reduced renin levels in the adult KO mice were largely due to a progressive increase in high BP, inhibiting renin synthesis and secretion from the juxtaglomerular (JG) cells of the kidneys (Shi et al., 2001; Pandey, 2008).

Studies from our laboratory further determined the quantitative contribution and mechanisms mediating the renal and cardiac hemodynamic responsiveness with decreasing or increasing numbers of *Npr1* gene copies (Shi et al., 2003; Vellaichamy et al., 2005, 2014; Das et al., 2010; Kumar et al., 2017; Periyasamy et al., 2019). Pure blood volume expansion of *Npr1* KO and gene-duplicated mice was used to determine the effect on renal blood flow (RBF), urine flow, glomerular filtration rate (GFR), and release of Na^+^ and K^+^ *Npr1* KO (0-copy), WT (2-copy), and gene-duplicated (4-copy) mice (Shi et al., 2003). Interestingly, hemodilution did not occur in whole blood, and plasma protein levels were not affected. Significant functional responses in RBF, Na^+^ excretion, and GFR were observed in *Npr1* WT (2-copy) and gene-duplicated (4-copy) mice but not in KO (0-copy) mice. Those previous studies indicated that mediating renal hemodynamic mechanisms and Na^+^ excretion in genetically modified *Npr1* mutant mice are heavily influenced by the ANP/NPRA axis (Shi et al., 2003). Both RBF and GFR were significantly reduced in *Npr1* 0-copy KO mice but increased in 4-copy gene-duplicated mice compared with 2-copy WT mice after pure blood volume expansion in these animals (Shi et al., 2003).

The ablation of *Npr1* is known to increase BP of homozygous KO mice, which was not affected by either minimal or high-salt diets. This suggested that the major effect of GC-A/NPRA may occur at the level of the vasculature and is likely independent of salt concentrations (Lopez et al., 1995). In contrast, later studies showed chronic elevation of BP in mice fed with high-salt diets when *Npr1* was disrupted (Oliver et al., 1998; Zhao et al., 2007, 2013). Studies with *Nppa* KO mice have also shown that defects in ANP synthesis may cause salt-sensitive hypertension in these mutant animals (John et al., 1995; Melo et al., 1998). The physiological and pathophysiological roles of the ANP/NPRA system in the regulation of high BP and renal and cardiac dysfunction have been demonstrated in genetic mouse models of both *Nppa* and *Npr1* (John et al., 1995; Lopez et al., 1995; Oliver et al., 1997, 1998; Pandey et al., 1999; Shi et al., 2003; Vellaichamy et al., 2005; Ellmers et al., 2007; Das et al., 2020; Subramanian et al., 2022). Genetic defects that reduce the activity of GC receptors may contribute to hypertension, leading to renal and cardiac dysfunction and congestive heart failure (CHF) in mutant animals (Shi et al., 2003; Ellmers et al., 2007; Vellaichamy et al., 2007; Pandey, 2008; Das et al., 2012; Vellaichamy et al., 2014; Subramanian et al., 2016; Das et al., 2020). The consequences of the ablation of NPs and NPRs in mice and the resultant specific phenotypes are presented in Table 1. The ablation of GC-A/NPRA greatly increases high BP, altered levels of renin, ANG II, other components of RAAS, and lethal vascular and cardiac disorders, resembling untreated hypertensive heart disease patients (Vellaichamy et al., 2007; Sezai et al., 2010; Zhao et al., 2013; Vellaichamy et al., 2014; Kumar et al., 2017; Periyasamy et al., 2019; Das et al., 2020; Sangaralingham et al., 2022), although *Npr1* gene duplication in mice showed significantly increased levels of cGMP and reduced BP with increasing *Npr1* gene copy numbers in a gene-dose-dependent manner (Oliver et al., 1998; Pandey et al., 1999; Shi et al., 2003; Zhao et al., 2007; Vellaichamy et al., 2014; Periyasamy et al., 2019; Das et al., 2020). ANP and BNP expression is increased in patients with cardiac hypertrophy and CHF; whether the protective role of NPs and the GC-A/NPRA system are activated by reducing the harmful effects of high BP caused by retention of sodium and fluid volume; by inhibiting the RAAS; or as a result of the hypertrophic and/or fibrotic remodeling in the kidney, heart, and vasculature (Cannone et al., 2019; Rubattu et al., 2019). The GC-A/NPRA system is crucial to the regulation of systemic and intracellular components of RAAS (Shi et al., 2001; Pandey, 2008). ANP-BNP concentrations are usually found at much higher levels in the cardiac tissues and plasma of CHF patients (Wei et al., 1993a,b; Chen and Burnett, 1998). Both *Nppa* and *Nppb* are overexpressed in the hypertrophied heart and may act as endogenous protective mechanisms against maladaptive cardiac hypertrophy and cardiovascular disorders (Masciotra et al., 1999; Knowles et al., 2001; Volpe, 2014; Reginauld et al., 2019). GC-A/NPRA seems to be downregulated in the severe chronic CHF patients. Genetic disruption of *Npr1* in mice increases the cardiac mass, leading to hypertrophic growth and disorders (John et al., 1995; Oliver et al., 1997; Vellaichamy et al., 2005; Ellmers et al., 2007; Zhao et al., 2013; Subramanian et al., 2016). There is evidence of a significant inverse relationship between left ventricular cardiac hypertrophy and myocardial *Nppa* and *Nppb* expression, suggesting that ANP and BNP expression play a protective role in cardiovascular disorders and CHF (Wei et al., 1993b; Masciotra et al., 1999; Kobayashi et al., 2012; Sangaralingham et al., 2022). It has been recently suggested that CNP is synthesized in the cardiac myocytes and endothelial cells also preserves heart structure–function and coronary reactivity (Moyes et al., 2020).

**Table 1:** Nomenclature, distribution, and gene-knockout phenotypes of natriuretic peptides and their specific receptors.

Peptide/Protein	Gene	Distribution	Gene-KO phenotype in mice	Literature cited
ANP	*Nppa*	Heart, kidney, brain,ovary, testis, and pituitary	Hypertension, hypertrophy, heart failure hypertension	John et al. (1995), Steinhelper et al. (1990), Lin et al. (1995), Melo et al. (1999), Tankersley et al. (2010)
BNP	*Nppb*	Brain and heart	Ventricular fibrosis, skeletal and vascular abnormalities	Tamura et al. (2000), Ogawa et al. (1994), Holditch et al. (2016)
CNP	*Nppc*	Vascular endothelium Brain, kidney, heart, and testis	Reduced long bone growth, dwarfism, abnormal chondrocyte growth	Chusho et al. (2001), Yasoda et al. (2004), Wang et al. (2007)
Guanylin		Intestine, kidney, brain, liver	Blockade of intestinal sodium and water excretion	Sangaralingham et al. (2022), Mattingly et al. (1994)
Uroguanylin		Intestine, kidney, Brain, liver	Disruption of intestinal fluid-ion balance, increase of BP	Sangaralingham et al. (2022), Mattingly et al. (1994)
GC-A/ NPRA	*Npr1*	Heart, kidney, adrenal glands, brain, lung, ovary, testis, thymus, vasculature, pituitary gland	Volume regulation, hypertension, cardiac hypertrophy and fibrosis, inflammation, cardiac overload, reduced testosterone, decreased estrogen	Ellmers et al. (2007), Oliver et al. (1997), Shi et al. (2003), Vellaichamy et al. (2005), Vellaichamy et al. (2014), Lopez et al. (1995), Das et al. (2010), Periyasamy et al. (2019), Zhao et al. (2007), Das et al. (2020), Subramanian et al. (2016), Reinhart et al. (2006), Nakanishi et al. (2005)
GC-B/NPRB	*Npr2*	Brain, heart, lung, ovary, pituitary gland, testis, cartilage, thymus, vasculature	Decreased adiposity, dwarfism, female sterility, seizures, vascular abnormalities	Tamura et al. (2004), Langenickel et al. (2006)
NPRC	*Npr3*	Kidney, brain, heart, intestine, liver, vasculature	Skeletal over-growth, bone deformation, long bone overgrowth	Jaubert et al. (1999), Matsukawa et al. (1999)
GC-C	*Gc-c*	Intestine, kidney, liver, brain	Resistance to exogenous *E. coli* heat-stable enterotoxin, disruption of intestinal fluid-ion homeostasis, unaffected BP	Schulz et al. (1997), Kuhn (2005)

ANP, atrial natriuretic peptide; BNP, brain natriuretic peptide; CNP, C-type natriuretic peptide; Nppa, coding for pro-atrial natriuretic peptide; Nppb, coding for pro-brain natriuretic peptide; Nppc, coding for pro-C-type natriuretic peptide. GC-A/NPRA, guanylyl cyclase/natriuretic peptide receptor-A; GC-B/NPRB, guanylyl cyclase/natriuretic peptide receptor-B; NPRC, natriuretic peptide receptor-C; Npr1, coding for GC-A/NPRA; Npr2, coding for GC-B/NPRB; Npr3, coding for NPRC; GC-C, guanylyl cyclase C.

## GC-A/NPRA regulation of metabolic and immunogenic responses

Decreased plasma levels of ANP are known to be associated with obesity, insulin resistance, energy, and glucose metabolism in human patients (Wang et al., 2007; Birkenfeld et al., 2008; Coue and Moro, 2016; Cannone et al., 2019). Thus, ANP-BNP/GC-A/NPRA signaling has been suggested as playing a role in the regulation of whole-body metabolism and diabetic conditions (Moro, 2013; Coue et al., 2015). The GC-A/NPRA system has been shown to enhance lipid mobilization, mitochondrial oxidative pathway, and fat oxidation, maintaining energy expenditure and fatty acid supply to cardiac and skeletal muscle metabolic processes (Tsukamoto et al., 2009; Birkenfeld et al., 2012; Engeli et al., 2012; Schlueter et al., 2014). On the other hand, NPRC exhibits a lipolytic effect of NPs (ANP, BNP). Mice challenged with low temperature were shown to have increased release of ANP but reduced levels of NPRC in both white and brown adipocytes (Sengenes et al., 2002; Bordicchia et al., 2012). In fact, insulin enhanced the NPRC expression in adipocytes in a glucose-dependent manner (Bordicchia et al., 2016). Defective or absent NPs/GC-A/NPRA signaling may promote maladaptive metabolic disorders that lead in decreased mitochondrial function, hyperglycemia, insulin resistance, and lipid accumulation, in turn, leading to hypertension and CVD in humans. At balance, the circulating concentrations of ANP may serve as a protective mechanism; however, low circulating levels of ANP may be related to cardiometabolic syndrome and an increased risk of diabetes (Pereira et al., 2015; Ceddia and Collins, 2020).

Circulating levels of harmful proinflammatory cytokines, including interleukin-1β (IL-1β), interleukin-6 (IL-6), and tumor necrosis factor alpha (TNF-α) trigger to the development of hypertension and renal and cardiovascular disorders in humans (Testa et al., 1996; Vanderheyden et al., 2005). Studies from our laboratory have suggested that proinflammatory cytokines promote the onset of hypertension, kidney disorders, CHF, and cardiac hypertrophy in experimental animal models (Das et al., 2010; Vellaichamy et al., 2014; Subramanian et al., 2016; Kumar et al., 2017; Gogulamudi et al., 2019; Periyasamy et al., 2019; Das et al., 2020; Subramanian et al., 2022). Enhanced activation of IL-1β, IL-6, and TNF-α, induce phenotypic changes in myocardial apoptosis, deposition of matrix proteins, myocyte hypertrophy, and contractile dysfunction (Thaik et al., 1995; Sekiguchi et al., 2004; Vellaichamy et al., 2007, 2014; Subramanian et al., 2016). Furthermore, our previous findings have shown that the NPs/GC-A/NPRA cascade inhibits inflammation and immunogenic responses in the heart and kidneys (Vellaichamy et al., 2005; Das et al., 2010; Vellaichamy et al., 2014; Subramanian et al., 2016; Kumar et al., 2017; Das et al., 2020). In macrophages, ANP blocked TNF-α production as well as TNF-α-induced adhesion molecule expression in endothelial cells (Tsukagoshi et al., 2001; Vollmer, 2005). We have found that genetically based differences in the expression levels of *Npr1* affect the contents of proinflammatory cytokine (Das et al., 2012; Zhao et al., 2013; Vellaichamy et al., 2014; Subramanian et al., 2016; Gogulamudi et al., 2019). Ablation of *Npr1* activates proinflammatory cytokine gene expression in addition to protein levels involved in cardiac hypertrophy and exaggerated ventricular remodeling, leading to cardiac dysfunction and CHF (Vellaichamy et al., 2014). However, in mice, cardiac proinflammatory cytokine expression and tissue remodeling were attenuated by *Npr1* gene duplication (Das et al., 2012; Vellaichamy et al., 2014; Subramanian et al., 2016; Kumar et al., 2017). *Npr1^−/−^* mutant mice expressed elevated levels of TNF-α, IL-6, interferon-gamma (IFN-γ), and transforming growth factor beta 1 (TGF-β1) mRNAs and proteins compared to *Npr1^+/+^* WT mice (Vellaichamy et al., 2014; Gogulamudi et al., 2019). In contrast, levels of IL-6, TNF-α, IFN-γ, and TGF-β1 were significantly reduced in *Npr1* gene-duplicated 3-copy and 4-copy mice compared to WT control mice.

The GC-A/NPRA-cGMP system exerts anti-inflammatory activity and inhibits the action of nuclear factor-kappa B (NF-κB) and proinflammatory cytokines (Tsukagoshi et al., 2001; Vollmer, 2005; Ellmers et al., 2007; Das et al., 2012; Pandey, 2018). Ablation of *Npr1* also enhances the expression of NF-kB, which seems to be involved in renal and cardiac disorders, fibrosis, hypertrophy, and extracellular matrix deposition (Vellaichamy et al., 2007, 2014; Kumar et al., 2017; Das et al., 2020). We speculated that activated NF-κB signaling triggers the production of proinflammatory cytokines, stimulating the development of tissue fibrosis and hypertrophy (Frantz et al., 2003; Purcell and Molkentin, 2003; Li et al., 2004; Vellaichamy et al., 2014; Subramanian et al., 2016; Das et al., 2020). Pathological forms of cardiac and renal remodeling, along with exacerbated disease conditions, are associated with the sustained activation of proinflammatory cytokines in experimental animals (Hirota et al., 1995; Baumgarten et al., 2002; Palmieri et al., 2002; Zhao et al., 2013; Vellaichamy et al., 2014; Pandey, 2021). We speculate that enhanced NPs and GC-A/NPRA signaling may have a protective effect on the heart and kidneys by inhibiting the expression of NF-κB, which is known to be a master regulator of proinflammatory cascade in health and disease.

## NPs and sodium-glucose cotransporters inhibitors mediate renal homeostasis and hypertension

NPs (especially ANP and BNP) play critical and pleotropic roles in modulating the release of Na^+^ and water from the kidneys thus decrease BP and prevent renal dysfunction (de Bold, 1985; Genest and Cantin, 1988; Brenner et al., 1990; Christensen, 1993; Levin et al., 1998; Pandey, 2005; Pandey, 2018). It was also reported that ANP restricted the intestinal absorption of water, Na^+^, and glucose (Martinez Seeber et al., 1986; O’Grady, 1989; O'Grady et al., 1989; Gonzalez Bosc et al., 1997). Subsequently, ^125^I-ANP binding studies demonstrated the presence of biologically active ANP receptor in the intestine jejunum (Bianchi et al., 1989). Further, it was suggested that the effect of ANP on the intestinal absorption of Na^+^ could be due to the blockade of sodium-glucose cotransporter (SGLT-2) in the intestine (Gonzalez Bosc et al., 2000). In fact, the greedy organs such as kidney and intestine play critical roles in contributing toward hypertension and type-2 diabetes melitus (T2 DM) (Itoh and Tanaka, 2022). These authors suggested that a new type of therapeutic intervention might be necessary for the multigate-greedy organ dysfunction. In the retrospect, SGLT-2 inhibitors might provide expected outcome for the treatment and prevention of both hypertension and T2 DM conditions.

SGLT-2 inhibitors effectively lower the glucose and Na^+^ levels, thus besides anti-diabetic effects, these agents may also exhibit renal and cardiac protective function (Maejima, 2019; Tsai et al., 2021). Major approved SGLT-2 inhibitors lower blood glucose, renal sodium, body weight and BP; however, improve adiposity and normalize serum uric acid and lipid levels (Alicic et al., 2018; Thomas and Cherney, 2018; Bailey, 2019; Tsai et al., 2021). It is stipulated that the renal-and cardio-protective mechanisms of SGLT-2 inhibitors include improved GFR, reduced inflammation, fibrosis, and sympathetic tone; as well as show enhanced mitochondrial biogenesis and function (Lee et al., 2019; Maejima, 2019; Zelniker and Braunwald, 2020; Margonato et al., 2021). It has been reported that SGLT-2 inhibitor treatment reduced the plasma ANP and BNP levels, thus reducing the cardiovascular risk in diabetic patients (Feng et al., 2020). However, N-terminal pro-BNP (NT-proBNP) is a better prognostic marker for prediction of heart failure in T2 DM (Cosson et al., 2009; Fraty et al., 2018).

## Conclusion and perspectives

Over the past four decades, new insights have emerged regarding the structure–function relationship, signaling cascades, and physiological functions of NPs and their receptor systems. Cellular, molecular, and genetic investigations have revealed the complex molecular signaling systems of GC-A/NPRA and the significance of the intracellular cGMP in receptor biology, physiology/pathophysiology of hypertension, renal and cardiovascular disorders, and neurotransmission in CNS. Researchers have developed novel tools to delineate the domain structures, signaling mechanisms, internalization and cellular distribution, and downregulation and desensitization of GC-A/NPRA. Gene-targeting strategies (knockout and duplication) and transgenic approaches in mice have helped to clarify the role of NPs and GC-A/NPRA in creating and intervening in disease states using genetically modified mouse models. Molecular, cellular, and integrative approaches *in vitro* and *in vivo* have provided revolutionary insights into the roles of NP/NPRA/cGMP signaling mechanisms in the regulation of hypertension and renal and cardiovascular diseases, paving the way toward discovery of effective therapeutics. The generation of the *Npr1*-deficient mouse model has contributed to advances in our understanding of the roles of GC-A/NPRA in the pathophysiology of hypertensive, endocrine, cardiovascular, and neurological dysfunction; however, the available models are still complex, partly due to the fact that the desired protein product is not present in the modified null animal models. As a result, the specific given phenotype may affect the degree of contribution of modifying gene products in disease conditions. In such situations, studies on haplotype or heterozygous mice might yield novel and useful information on disease states in animal models similar to those in genetic polymorphisms in human patients.

Despite the ample progress, there is still much to discover regarding the novel mechanisms of GC-coupled NP receptors and their ligands in relation to receptor activation mechanisms, cellular signaling, molecular modeling of structural determination, and physiological and pathophysiological functions. The mechanisms underlying ligand-dependent receptor activation and transmembrane signal transduction, which render the generation of second messenger cGMP, are not yet fully understood, nor are the molecular events that terminate the activated flow of receptor-mediated signal in the target cells. A more thorough understanding of the roles of NPs, GC-A/NPRA, and cGMP-specific signaling mechanisms in disease states is still needed. The identification and delineation of discrete switch points in signal transduction of GC-A/NPRA that elicit certain responses, such as renal function, cardioprotection, neurotransmission, and directional functions in reducing adverse BP and cardiovascular events, will provide new opportunities to intervene and deter renal, cardiac, endocrine, vascular, and neurological disorders. Future investigations should lead to exciting and innovative research strategies and new discoveries in the field of NPs and GC-A/NPRA/cGMP signaling mechanisms toward the prevention, diagnosis, and treatment of hypertension and renal and cardiovascular diseases.

## Author contributions

KNP conceived and wrote the manuscript, and completed the composition and references.

## Funding

The research in the author’s laboratory is supported by the grants from the National Institutes of Health (HL062147 and DK133833).

## Conflict of interest

The author declares that the research was conducted in the absence of any commercial or financial relationships that could be construed as a potential conflict of interest.

## Publisher’s note

All claims expressed in this article are solely those of the authors and do not necessarily represent those of their affiliated organizations, or those of the publisher, the editors and the reviewers. Any product that may be evaluated in this article, or claim that may be made by its manufacturer, is not guaranteed or endorsed by the publisher.

## Glossary

**Table:**

NPs	natriuretic peptides
ANF/ANP	atrial natriuretic factor/peptide
ANP, BNP, and CNP	atrial, brain, and C-type NPs
GC-A/NPRA	guanylyl cyclase/natriuretic peptide receptor-A
*Npr1*	Encoding GC-A/NPRA
BP	blood pressure
BNP	Brain natriuretic peptide
CNP	C-type natriuretic peptide
DNP	*Dendroaspis* natriuretic peptide or D-type NP
URO	urodilatin
GC-A/NPRA	Guanylyl cyclase NP receptor-A
GC-B/NPRB	GC/NP receptor-B
NPRC	NP receptor-C
PDs	phosphodiesterases
CNGs	cyclic-nucleotide gated ion channels
VSMCs	vascular smooth muscle cells
c-ANF or AP I	truncated ANF/ANP
MDCK	Maiden-Darby canine kidney
NPRs	NP receptors
CNS	central nervous system
LBD	ligand-binding domain
TD	transmembrane domain
protein-KHD	protein kinase-like homology domain
DD	dimerization domain
GCCD	guanylyl cyclase catalytic domain
RTKs	receptor tyrosine kinases
ACII C2	adenylyl cyclase II C2
WT	wild-type
JMHR	juxtamembrane hinge region
IP_3_	inositol trisphosphates
RIMCDs	renal inner medullary collecting duct cells
RMECs	renal medullary epithelial cells
PKC	protein kinase C
EGF	epidermal growth factor
PDGF	platelet-derived growth factor
ST	heat-stable enterotoxin
MMCs	mouse mesangial cells
miR	micro-RNA
MDC	monodansylcadaverine
CPZ	chlorpromazine
KO	knockout
JG	juxtaglomerular cells
RBF	renal blood flow
GFR	glomerular filtration rate
CHF	congestive heart failure
IL-1β	interleukin-1β
IL-6	interleukin-6
TNF-α	tumor necrosis factor alpha
IFN-γ	interferon-gamma
TGF-β1	transforming growth factor beta 1
NT-proBNP	N-terminal pro-BNP
